# Inter‐individual variability in physiological adaptations during heat acclimation in adults: Contributions of body mass index and body size

**DOI:** 10.14814/phy2.70713

**Published:** 2026-01-16

**Authors:** Shaun C. Brazelton, Nisha Charkoudian, Karleigh E. Bradbury, Roy M. Salgado, Gabrielle E. W. Giersch

**Affiliations:** ^1^ Thermal and Mountain Medicine Division US Army Research Institute of Environmental Medicine Natick Massachusetts USA; ^2^ Oak Ridge Institute for Science and Education Oak Ridge Tennessee USA

**Keywords:** body mass index, environmental physiology, thermoregulation

## Abstract

Heat acclimation refers to the physiological adaptations that occur during repeated heat exposures, ultimately reducing thermal and cardiovascular strain in the heat. It is unknown whether body mass index (BMI) influences an individual's ability to adapt during heat acclimation, which was tested in the present analysis. Forty‐two healthy adults (16F; age: 23 ± 5 years) underwent 8 days of treadmill walking (5 km·h^−1^) in the heat (40°C, 40% RH). Groups were compared based on BMI (<25 and >25). We measured core temperature (T_C_), heart rate (HR) and whole‐body sweating rate (WBSR) on days 1, 4 and 8. The BMI <25 group showed decreases in peak T_C_ (D1: 38.62°C ± 0.58°C, D4: 38.27 ± 0.38, D8: 38.10 ± 0.32; *p* ≤ 0.018). The BMI >25 group showed a reduction in peak T_C_ only on Day 8 (38.35 ± 0.45) compared to Day 1 (38.54 ± 0.53, *p* = 0.019). Peak T_C_ was lower in the BMI <25 group compared to the BMI > 25 group on Day 8 only (*p* = 0.042). HR decreased and WBSR increased over time, with no difference between groups (*p* > 0.05). The BMI <25 group showed greater reductions in peak T_C_ from D1 to D8 than the BMI >25 group (*p* = 0.010). These data suggest that individuals with BMI >25 may have attenuated T_C_ adaptations to heat acclimation compared to individuals with BMI <25.

## 1 INTRODUCTION

Exercise in the heat is associated with performance decrements (Cheuvront et al., 2010) and clinical outcomes ranging from heat exhaustion to exertional heat stroke, which can be fatal (Bouchama et al., 2022; Roberts et al., 2021). Heat acclimation (HA) is the gold standard pre‐emptive countermeasure for individuals who will be exercising in the heat (Roberts et al., 2021), and leads to adaptations including decreased core temperature (Tc) and heart rate (HR) and an increase in sweating (Périard et al., 2015, 2021; Sawka et al., 2011). Several factors have been investigated for their influence on HA responses. Recent research observed body mass and body surface area to mass ratio (BSA:mass) had a significant influence on cardiovascular and sweating responses to HA using isothermic protocols (Alkemade et al., 2021). These findings suggest body size may independently influence thermoregulatory responses to HA. Given the practicality of controlled work rate HA protocols (keeping exercise intensity consistent over the protocol), it is important to understand the potential influence of physical factors on adaptation within this context.

Individual physical characteristics, such as body fat percentage (BF%), fitness status, and body surface area to mass ratio (BSA:mass), are known to independently impact body temperature during exercise heat stress and heat illness risk (Bedno et al., 2014; Selkirk & McLellan, 2001; Taylor et al., 2024). A commonly used metric to evaluate physical characteristics is body mass index (BMI). In addition to being widely utilized in a population setting, there is significant research evaluating BMI and the related population cutoffs that are easily generalizable to the greater population (Eijsvogels et al., 2011; Gonze et al., 2021; Zierle‐Ghosh & Jan, 2025). Although BMI is limited in predicting physiological or metabolic health at the individual level (since it is not a measure of body composition), it is widely used to evaluate the overall health of populations. Notably, BMI is increasing in the general United States population (Banas et al., 2024), and the United States Army has seen increases in obesity (BMI > 30) in recent years (Defense Centers for Public Health, 2022). This trend is relevant because individuals with increased BMI may experience altered responses to exercise heat stress. Previously, male United States Marine Corps recruits with a higher BMI showed a higher risk for developing exertional heat illness during training compared to matched controls (Gardner et al., 1996). Work from our laboratory also showed that each unit increase in BMI led to an increase in relative risk for exertional heat stroke risk by ~3% in United States Army Soldiers (Giersch et al., 2023), and that the risk was largely driven by biophysical characteristics, specifically BSA:mass (Taylor et al., 2024). Another recent study of United States Army enlistees showed an increased risk of severe exertional heat illness for individuals with a BMI classification as overweight (>25) (Kazman et al., 2025). Additionally, Akavian and colleagues recently reported that individuals categorized as “heat intolerant” during testing following an exertional heat illness had a higher mass, BMI, higher BF%, and lower BSA:mass compared to those that had thermoregulatory responses consistent with the “heat tolerant” category (Akavian et al., 2025). Taken together, these findings suggest a potential increase in heat strain in individuals with higher BMI, though this has not yet been verified with laboratory‐based studies. This increased heat strain, when below levels that contribute to heat‐related illness, could possibly enhance adaptation responses whereby greater increases in body temperature may elicit greater magnitude or time course of adaptation.

The challenges of exercise in the heat are not limited to body temperature regulation, as these stressors concurrently impose challenges on other physiological systems, including the cardiovascular system. Cardiovascular strain during heat stress is thought to be related to the competition for blood flow to working muscle during exercise and blood flow to the skin for thermoregulation (González‐Alonso et al., 2008; Rowell, 1974). There is evidence that individuals with higher BMI experience greater cardiovascular strain during work in hot and humid conditions compared to individuals with lower BMI (Dehghan et al., 2013). However, it is unclear if this finding is related specifically to body size, fitness, or body composition. Relative to body composition, individuals with higher BF% also appear to experience greater cardiovascular strain during exercise heat stress (Selkirk & McLellan, 2001). Cardiovascular strain is increased in individuals with lower fitness levels (Cheung & McLellan, 1998), which is relevant as estimated maximal oxygen uptake is lower as BMI increases (Chen et al., 2017).

Currently it is unknown whether BMI influences physiological adaptation during controlled work rate heat acclimation and if any relationship between BMI and hyperthermia (increased T_C_) may be influenced by acclimation status. Therefore, the purpose of the present analysis was twofold: (1) To test whether BMI influences the magnitude of adaptation over the course of heat acclimation; and (2) To evaluate the relationship between BMI and hyperthermia in both an unacclimated and a heat acclimated state. Given that individuals with higher BMI and lower BSA:mass are at a greater risk for heat‐related illness, we hypothesized that individuals with higher BMI would have greater thermal strain during exercise in the heat. The magnitude of adaptation during a heat acclimation protocol is related to the thermal strain experienced by the individual, with greater thermal strain leading to greater adaptation (Fox et al., 1963). Based on this increased thermal strain (i.e., T_C_), we also hypothesized that the higher BMI group would have a larger magnitude of adaptation. This work builds on the previous work from our laboratory that evaluated the influence of body size on heat illness risk (Giersch et al., 2023; Taylor et al., 2024).

## 2 METHODS

### 2.1 Volunteers

The present report represents a secondary analysis of data from 42 total volunteers (Table 1) from two previous studies (Giersch et al., 2025; Salgado et al., 2020). Both studies were approved by the Institutional Review Board of the United States Army Medical Research and Development Command and all volunteers provided written, informed consent after all study procedures and possible risks were described (Protocol numbers: M‐10715, M‐10929). Investigators adhered to policies for the protection of human subjects as prescribed in U.S. Army Regulation 70–25 and U.S. Army Medical Research and Development Command Regulation 7–25. The research was conducted in adherence with the provisions of Code 45 of Federal Regulations Part 46. Investigations also adhered to all facets of the *Declaration of Helsinki* (Clinicaltrials.gov registration no. NCT05292170), except for registration in a database for Salgado et al. (2020).

**TABLE 1:** Volunteer characteristics.

	BMI < 25 (*n* = 19 (7 F))	BMI > 25 (*n* = 23 (9 F))	Total (*n* = 42 (16 F))
Age (year)	21 ± 3	25 ± 6	23 ± 5
Height (cm)	170.9 ± 10.4	170.4 ± 10.3	170.6 ± 10.2
Body mass (kg)	66.72 ± 9.06	83.23 ± 13.27*	75.76 ± 14.13
BMI (kg ·m^−2^)	22.77 ± 1.56	28.52 ± 2.15*	25.92 ± 3.48
Body fat (%)	24.1 ± 7.8	32.2 ± 7.0*	28.5 ± 8.4
BSA (m^2^)	1.7984 ± 0.1759	1.9503 ± 0.1673*	1.8816 ± 0.1857
BSA:mass (m^2^·kg^−1^)	0.0271 ± 0.0014	0.0237 ± 0.0018*	0.0252 ± 0.0024
V̇O_2peak_ (mL·kg^−1^·min^−1^)	43.60 ± 6.19	38.69 ± 5.74*	40.91 ± 6.37
*Race*/*Ethnicity*			
White/Hispanic or Latino	3 (7.14%)	9 (21.43%)	12 (28.57%)
White/Not Hispanic or Latino	9 (21.43%)	5 (11.90%)	14 (33.33%)
Black/Not Hispanic or Latino	2 (4.76%)	3 (7.14%)	5 (11.90%)
Asian/Not Hispanic or Latino	3 (7.14%)	1 (2.38%)	4 (9.52%)
American Indian or Alaska Native/Not Hispanic or Latino	0 (0%)	1 (2.38%)	1 (2.38%)
Native Hawaiian or Pacific Islander/Hispanic or Latino	1 (2.38%)	0 (0%)	1 (2.38%)
Other/Hispanic or Latino	1 (2.38%)	2 (4.76%)	3 (7.14%)
Other/Not Hispanic or Latino	0 (0%)	2 (4.76%)	2 (4.76%)

*Note*: Data are presented as mean ± SD. Race and ethnicity self‐reported by volunteers; other includes volunteers that did not report race or reported multiple races. Race and ethnicity presented as *n* (%).

Abbreviations: BMI, body mass index; BSA, body surface area; V̇O_2peak_, peak oxygen consumption.

* *p* < 0.05 compared with BMI < 25.

### 2.2 Baseline visit

During a baseline visit, volunteers self‐reported age, sex, race, and ethnicity using a background questionnaire. Height was measured using a stadiometer (Seca 213, Seca corporation, Hanover, MD, USA) and a private nude body mass was measured (Adam Equipment, CPWPlus‐200, Oxford, CT, USA). Additionally, BF% was measured using dual energy X‐ray absorptiometry (DEXA; GE Lunar iDXA, GE Healthcare, Madison, WI, USA). Body surface area (BSA) was calculated (Du Bois & Du Bois, 1989) (*n* = 13) or measured using a 3‐D scan (SS20, SizeStream, Cary, NC, USA) (*n* = 29). To maximize ecological validity, testing in women was not restricted by menstrual cycle phase or contraceptive use.

### 2.3 V̇O_2peak_ assessment

Peak oxygen consumption (V̇O_2peak_) was measured prior to the acclimation protocol using an incremental cycle protocol (*n* = 13) or a graded treadmill exercise protocol (*n* = 29) and a closed‐circuit spirometry system (TrueOne 2400, ParvoMedics, Sandy, UT, USA). The cycling protocol began at 75 W (50 W for one volunteer) for 3 min and the workload was increased by 25 W every minute until volitional exhaustion (until cadence of 65–75 revolutions per minute could not be maintained) (Salgado et al., 2020). The treadmill protocol used a fixed grade with increases in speed (0.8 km·h^−1^) every 3 min until volitional exhaustion (Giersch et al., 2025).

### 2.4 Heat acclimation

Volunteers completed 8 days of treadmill walking (5 km·h^−1^, 2% grade) in a hot climatic chamber (40°C, 40% RH, ~4.8 km·h^−1^ air velocity) for 120 min wearing shorts, a t‐shirt, sports bra (female only), socks and sneakers. Prior to beginning exercise, hydration was checked via urine specific gravity (USG; REF312ATC, General Tools and Instruments, Secaucus, NJ, USA; PEN‐USG 3741, Atago, Bellevue, WA, USA). To ensure euhydration prior to the start of exercise heat stress, for the first study (Salgado et al., 2020), if USG≥1.020, an additional 250 mL of water was provided with a standardized breakfast prior to the start of exercise; for the second study included (Giersch et al., 2025) if USG>1.025, 500 mL of water was provided prior to the start of exercise. To maintain euhydration throughout exercise, volunteers were given 200 mL of room temperature water every 20 min (1.2 L total). T_C_ was measured via a wireless telemetry pill (eCelsius, BodyCap, France; Mini Mitter, Bend, OR, USA) either ingested on the night prior to the trial (*n* = 13) (Salgado et al., 2020) or inserted as a suppository (*n* = 29) (Giersch et al., 2025) and recorded every 5 min during exercise. HR was measured with a chest‐worn strap (Polar A3, Polar Electro, Woodbury, NY, USA) and recorded every 5 min during exercise. Skin temperature (T_Sk_) was measured for a subset of volunteers (*n* = 26) with wireless temperature sensors (iButton, Analog Devices, USA) affixed to four sites (chest, deltoid, thigh and calf). Nude body mass was measured privately immediately before and after exercise. Water consumed and any urine produced during the trial were weighed to determine fluid consumption and urine output. All visits occurred at the same time of day (± 1 h) between October and May in Natick, MA to limit the effects of circadian rhythm and seasonal acclimatization on thermoregulation. Trials were terminated when one of four criteria were met: (1) 120 min of walking completed, (2) T_C_ ≥ 39.50, (3) signs of heat illness, or (4) volunteer requested to stop.

### 2.5 Calculations

BMI was calculated from the height and mass measured at the baseline visit (kg·m^−2^). BSA:mass (m^2^·kg^−1^) was also calculated using the baseline visit mass and the BSA values either measured (*n* = 26) or calculated (*n* = 13). Peak T_C_ and peak HR were defined as the highest recorded T_C_ and HR during exercise, respectively. The change in T_C_ during exercise (Δ T_C_) was calculated as the change from the T_C_ at the start of exercise to the T_C_ at the end of exercise. Mean weighted skin temperature (MWT_Sk_) was calculated with the following equation (Ramanathan, 1964):

$${\mathrm{MWT}}_{\mathrm{Sk}}=0.3T_{\text{Chest}}+0.3T_{\text{Deltoid}}+0.2T_{\text{Thigh}}+0.2T_{\text{Calf}}$$

In the subset of volunteers that had skin temperature measured (*n* = 26), the highest MWT_Sk_ recorded was then analyzed as peak MWT_Sk_. We calculated core‐to‐skin temperature gradient as the difference between peak T_C_ and peak MWT_Sk_ (Cheuvront et al., 2003) for a given day. Whole body sweating rate (WBSR) was calculated from the change in nude body mass from pre‐ to post‐exercise, accounting for fluid consumption and urine output. WBSR relative to BSA was included in this investigation given the differences in body size and BSA between groups.

### 2.6 Statistical analysis

As this was a secondary analysis, no a priori power analysis was conducted. Volunteers were grouped above and below the overweight classification threshold of BMI (25 kg·m^−2^). Data were assessed for normality via Shapiro–Wilk tests and visual inspection of quantile‐quantile plots. The controlled work rate protocols that were utilized in the studies in the present analysis allow for comparison across days of the acclimation protocol. Group characteristics and the change in peak T_C_ from Day 1 to Day 8 were compared via Welch's *t*‐tests or a Mann–Whitney *U* test when the assumption of normality was violated (i.e., age, BSA). The impacts of BMI group on physiological measures (i.e., peak T_C_, ΔT_C_, peak HR, WBSR, peak MWT_Sk_ and core‐to‐skin temperature gradient) on Day 1, Day 4, and Day 8 of heat acclimation were assessed by fitting a mixed‐effects model. Where appropriate, Tukey's post‐hoc correction was applied to pairwise comparisons. To assess the relationship between BMI and peak T_C_, Pearson product correlation coefficients were calculated on Day 1 and Day 8. In the case that correlations were significant, a simple linear regression with 95% CI bands was performed.

Two volunteers did not complete 8 days of heat acclimation and thus only have data on Days 1 and 4. Due to an issue with the pill reading at the beginning of exercise on Day 1 for 1 volunteer, peak T_C_ data is included but ΔT_C_ is excluded. One volunteer had all data from Day 1 excluded due to exiting the chamber to be evaluated for a headache prior to resuming exercise. One volunteer ingested the T_C_ pill on Day 1 and Day 8 but inserted the pill as a suppository on Day 4 due to an issue measuring the ingested pill. WBSR data is missing for four trials (*n* = 4 volunteers, one trial each) due to exclusion, as mentioned above, and missing body mass measurements.

Data were analyzed using Prism v10.1.2 (GraphPad PRISM, La Jolla, CA, USA) and R (Version 4.4.1; R foundation for Statistical Computing, Vienna, Austria). Statistical significance was set at *p* ≤ 0.05. Data are reported as mean ± SD unless otherwise stated.

## 3 RESULTS

**FIGURE 1:**
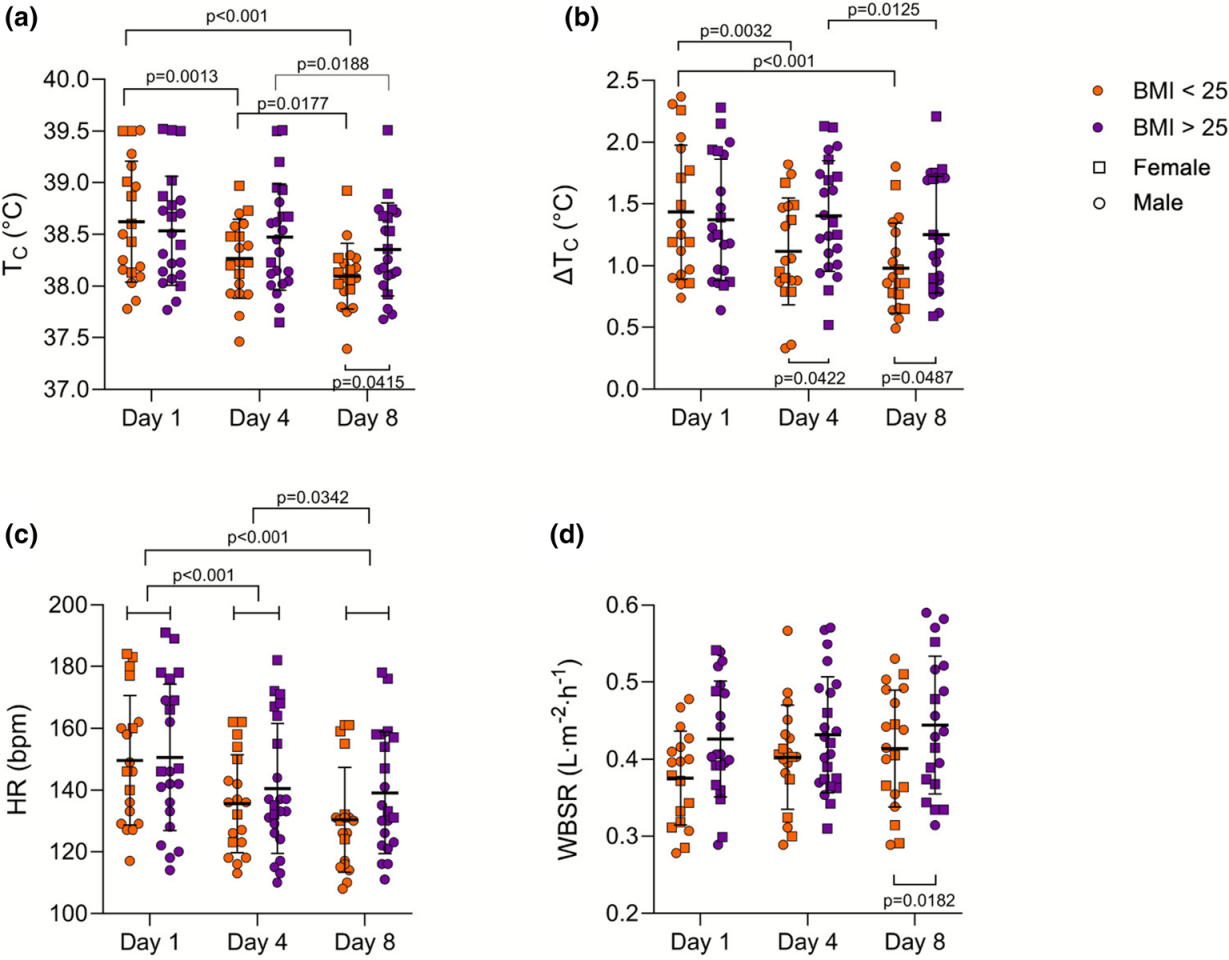
Physiological measures (a: Peak T_C_, b: Delta T_C_, c: Peak HR, d: WBSR) during exercise on Day 1, Day 4, and Day 8 in BMI < 25 and BMI > 25 groups. Data are shown as mean ± SD with individual data. Male participants are represented by circles and female participants are represented by squares. Data were analyzed with a mixed‐effects model with Tukey's post‐hoc correction for pairwise comparisons where appropriate. Sample sizes shown are Panel (a) (D1: *N* = 41, D4: *N* = 42, D8: *N* = 40), Panel (b) (D1: N = 40, D4: *N* = 42, D8: *N* = 40), Panel C (D1: *N* = 41, D4: *N* = 42, D8: *N* = 40), and Panel D (D1: *N* = 39, D4: *N* = 42, D8: *N* = 39).

**FIGURE 2:**
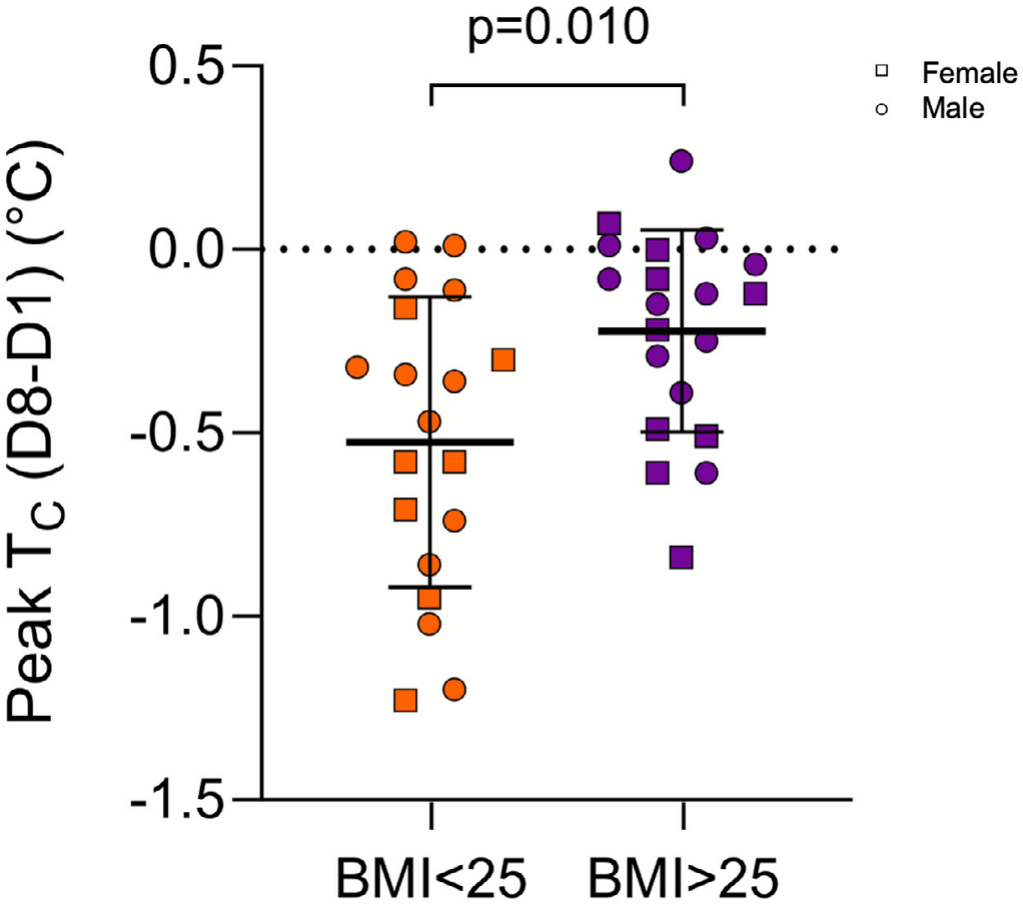
Core temperature adaptation as shown by the difference in peak T_C_ between Day 8 and Day 1 for BMI < 25 (*n* = 19) and BMI > 25 (*n* = 20) groups. Data are shown as mean ± SD with individual data. Male participants are represented by circles and female participants are represented by squares. Data were analyzed with Welch's *t*‐test.

Volunteer characteristics are presented in Table 1. The BMI < 25 and BMI > 25 groups did not differ in age or height (*p* > 0.05). The BMI > 25 group had a higher mass (*p* < 0.001), BMI (*p* < 0.001), BF% (*p* = 0.001), BSA (*p* = 0.009) and a lower BSA:mass (*p* < 0.001) and V̇O_2peak_ (*p* = 0.012) compared to the BMI < 25 group. Baseline T_C_ was unaffected by HA in both BMI groups (BMI < 25: Day 1–37.17 ± 0.27, Day 4–37.09 ± 0.22, Day 8–37.08 ± 0.20, *p* > 0.05; BMI > 25: Day 1–37.10 ± 0.26, Day 4–37.06 ± 0.31, Day 8–37.08 ± 0.23, *p >* 0.05). Figure 1 shows group mean and individual data for Tc, HR and WBSR between groups and across days of HA. Peak T_C_ (Figure 1a) showed an effect of Day (*p* < 0.001) and an interaction between Day and Group (*p* = 0.003). The BMI < 25 group had a lower peak T_C_ on Day 8 (38.10°C ± 0.32°C) compared to Day 1 (38.62°C ± 0.58°C, *p* < 0.001) and Day 4 (38.27°C ± 0.38°C, *p* = 0.018) and also on Day 4 compared to Day 1 (*p* = 0.001). The BMI > 25 group showed a reduction in peak T_C_ on Day 8 (38.35 ± 0.45) compared to Day 1 (38.54 ± 0.53, *p* = 0.019). Peak T_C_ was lower in the BMI < 25 group compared to the BMI > 25 group on Day 8 (*p* = 0.042). In the subset of volunteers that had peak T_C_ data on D1 and D8 (*n* = 39), the reduction in peak T_C_ from D1 to D8 was greater in the BMI < 25 group (−0.53°C ± 0.40°C) than in the BMI > 25 group (−0.22°C ± 0.27°C, *p* = 0.010) as shown in Figure 2.

Delta T_C_ (Figure 1b) showed an effect of Day (*p* < 0.001) and an interaction between Day and Group (*p* = 0.005). The BMI < 25 group had a reduction in Delta T_C_ on both Day 4 (1.12°C ± 0.43°C, *p* = 0.003) and Day 8 (0.98°C ± 0.36°C, *p* < 0.001) compared to Day 1 (1.43°C ± 0.54°C). The BMI > 25 group had a lower Delta T_C_ on Day 8 (1.25°C ± 0.47°C) compared to Day 4 (1.40°C ± 0.45°C, *p* = 0.013) with no difference from Day 1 (1.37°C ± 0.49°C, *p* > 0.05). The BMI < 25 group had a lower Delta T_C_ on Day 4 (*p* = 0.042) and Day 8 (*p* = 0.049) compared to the BMI > 25 group.

Peak HR (Figure 1c) showed an effect of Day (*p* < 0.001) with a reduction in peak HR when groups were combined on Day 8 (135 ± 19 bpm) compared to both Day 1 (150 ± 22 bpm, *p* < 0.001) and Day 4 (138 ± 19 bpm, *p* = 0.034), and on Day 4 compared to Day 1 (*p* < 0.001). There was no effect of Group and no interaction between Day and Group for peak HR (*p* > 0.05).

WBSR (Figure 1d) showed an effect of Day (*p* = 0.015), but no effect of Group or interaction between Day and Group (*p* > 0.05). WBSR was greater on Day 8 (0.4294 ± 0.0832 L·m^−2^·h^−1^) than Day 1 (0.4028 ± 0.0727 L·m^−2^·h^−1^, *p* = 0.044). Day 4 (0.4185 ± 0.0725 L·m^−2^·h^−1^) did not differ from Day 1 or Day 8 (*p* > 0.05).

**FIGURE 3:**
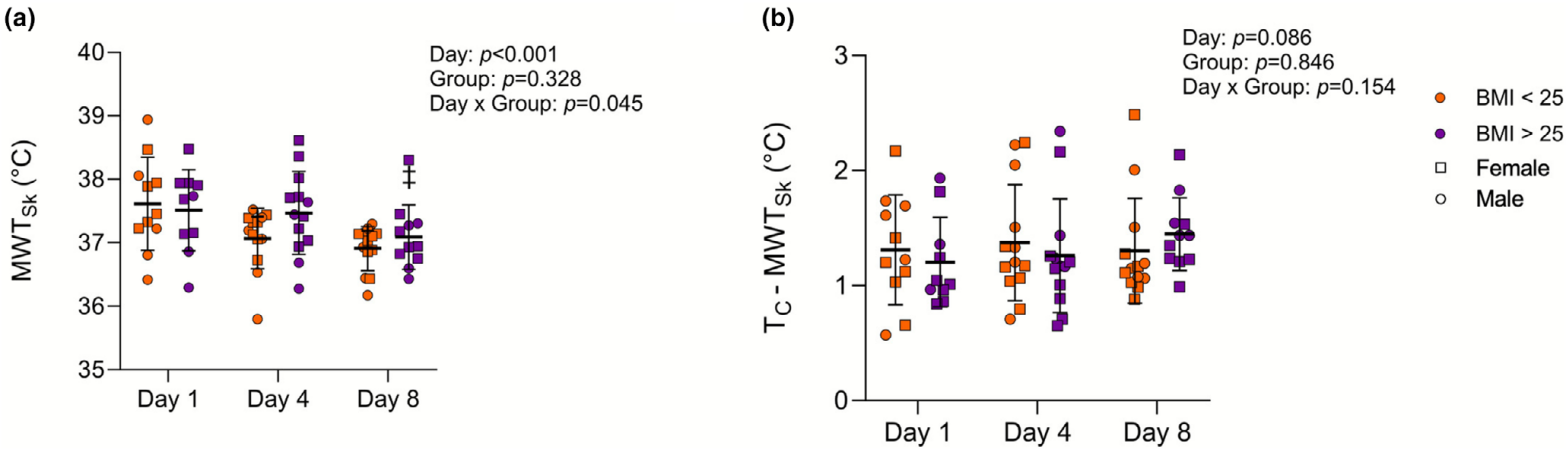
peak MWT_Sk_ (a) and core‐to‐skin temperature gradient (peak T_C_—peak MWT_Sk_) (b) during exercise on Day 1, Day 4, and Day 8 in BMI < 25 and BMI > 25 groups. Data are shown as mean ± SD with individual data. Male participants are represented by circles and female participants are represented by squares. Data were analyzed with a mixed‐effects model with Tukey's post‐hoc correction for pairwise comparisons where appropriate. Sample sizes were the same for both Panel (a) and Panel (b) (D1: *N* = 21, D4: *N* = 26, D8: *N* = 24).

A portion of the peak MWT_Sk_ data in this investigation have been previously reported grouped by sex (Giersch et al., 2025). For the purpose of this analysis, these data have been subdivided based on BMI (Figure 3a, BMI < 25: *n* = 13, 9F; BMI > 25: *n* = 13, 7F). There was an effect of Day (*p* < 0.001) and an interaction between Day and Group (*p* = 0.045). The BMI < 25 group had a lower peak MWT_Sk_ on Day 8 (36.91°C ± 0.35°C) compared to Day 1 (37.61°C ± 0.73°C, *p* = 0.003) and also on Day 4 (37.07°C ± 0.48°C) compared to Day 1 (*p* = 0.007). The BMI > 25 group had a lower peak MWT_Sk_ on Day 8 (37.09°C ± 0.51°C) compared to Day 4 (37.47°C ± 0.65°C, *p* = 0.010) with no differences from Day 1 (37.51°C ± 0.64°C, *p* > 0.05). The groups were not different from each other on any given day (*p* > 0.05).

**FIGURE 4:**
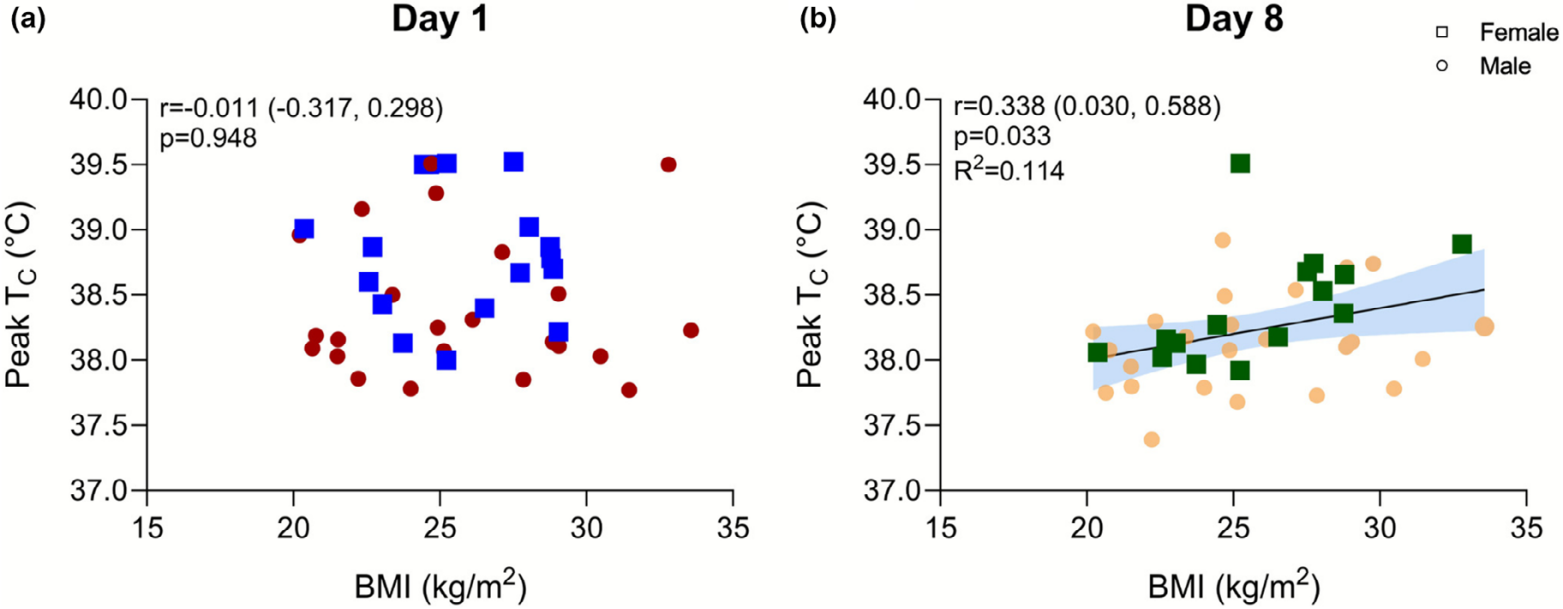
Pearson correlation coefficients between BMI and peak T_C_ on Day 1 and Day 8. Correlation coefficient with 95% CI and *p*‐value reported within figure for each day. A simple linear regression was performed on Day 8 due to the statistically significant correlation. The coefficient of determination is reported and the linear regression with 95% CI bands is shown in the figure. Sample sizes shown are *n* = 41 for Panel (a) and *n* = 40 for Panel (b). Male participants are represented by circles and female participants are represented by squares.

Core‐to‐skin temperature gradient (Figure 3b) showed no effect of Day (*p* = 0.086) or Group (*p* = 0.846) and no interaction between Day and Group (*p* = 0.154) for the subset of volunteers where skin temperature was measured (*n* = 26). There was no correlation between BMI and peak T_C_ on Day 1 (*r* = −0.011, *p* = 0.948), while there was a significant correlation on Day 8 (*r* = 0.338, *p* = 0.033) (Figure 4a,b). A simple linear regression with 95% CI bands is shown for Day 8 (Figure 4b) since there was a significant correlation.

## 4 DISCUSSION

The primary finding in the present analysis was that healthy young adults with higher (>25) BMI showed less T_C_ adaptation to an eight‐day heat acclimation protocol compared to lower (<25) BMI individuals. This contrasted with our hypothesis that people with higher BMI would experience greater thermal strain leading to greater T_C_ adaptation over the course of heat acclimation. The groups showed similar T_C_ responses on the first day of the protocol, while on the eighth day, the higher BMI group had a higher peak T_C_ and a greater increase in T_C_ during exercise compared to the BMI < 25 group (Figure 1a,b). While not a strictly physiological measure, BMI provides valuable insight into the role of body size on heat acclimation. Specifically, the thermophysiological factors of body size (BSA:mass) are very likely the key contributors to these findings.

The finding that larger individuals showed a reduced or delayed T_C_ adaptation may help partially explain epidemiological data that links a high BMI to a greater risk of exertional heat illness (Gardner et al., 1996; Kazman et al., 2025) and exertional heat stroke (Giersch et al., 2023). The slower T_C_ adaptation in the high BMI group led to higher T_C_ on Day 8, which may represent a limitation in the benefits of heat acclimation (e.g., EHI prevention) in larger individuals. However, there are additional factors associated with larger body size that may also contribute to this risk, such as lower fitness and higher BF% (Bedno et al., 2014). Lower fitness is an important factor in this analysis given the absolute exercise intensity of the controlled work rate protocol. Thus, individuals with lower fitness would have been exercising at a greater percentage of their relative VO_2max_. This would increase their cardiovascular responses to the exercise activity and potentially influence their thermoregulatory responses. To further support our finding that the higher BMI group showed attenuated T_C_ adaptation, we found a positive correlation between BMI and peak T_C_ on Day 8 (Figure 4) while there was no correlation on Day 1. This association, while not causative, suggests that larger individuals may experience higher T_C_ than smaller individuals when acclimated, possibly related to differences in magnitude of adaptation, as observed in our group comparison. Taken together, this may suggest that larger individuals with a higher BMI may benefit from a tailored heat acclimation approach (e.g., longer heat acclimation protocol duration) to induce further core temperature adaptations that may lower the risk for developing heat illnesses.

In the present analysis, the low BMI group showed decreases in peak MWT_Sk_ beginning on Day 4 while the high BMI group only showed a decrease on Day 8. The differential MWT_Sk_ findings between groups may be related, in part, to differences in BF% between groups (Havenith et al., 1995), although the independent effects of BF% on MWT_Sk_ are not clearly established across different exercise and heating paradigms (Morrissey et al., 2021). The adaptation time course for peak MWT_Sk_ within each group is similar to what was observed with peak T_C_ responses, with the higher BMI group possibly showing a delayed or attenuated adaptation. It is possible that the similar findings between T_C_ and MWT_Sk_ may also be related to the lower BSA:mass in the high BMI group, as differences in BSA:mass have previously been shown to explain thermoregulatory differences related to body size where individuals with higher BSA:mass (i.e., smaller individuals) may have a greater heat dissipation capacity relative to their heat production at a given workload (Cramer & Jay, 2014).

Our results regarding T_C_ adaptations were in opposition to our hypothesis that larger individuals would have a larger magnitude of adaptation due to higher thermal strain on individual days. We therefore wanted to evaluate how body size may have influenced the differences we observed. Previously, it has been proposed that a larger body size may act as a heat sink during heat stress (Havenith et al., 1998). However, this may not have been the case in the present analysis, as peak T_C_ was similar between groups on Days 1 and 4 and higher in the high BMI group on Day 8, indicating that the high BMI group did not experience reduced thermal strain. Within the subgroup that had T_Sk_ measured, we evaluated core‐to‐skin temperature gradient and found no differences based on group or day and no interaction between group and day. This is not necessarily surprising given the similarities between T_C_ and MWT_Sk_ in adaptation time course within the groups. This may suggest that there were no differences in skin blood flow between groups or over time, as core‐to‐skin gradient has been previously related to skin blood flow (Pergola et al., 1996; Rowell, 1986). However, skin blood flow was not directly measured in either investigation used in this secondary analysis, so this remains an open question. Additionally, evaporation of sweat can also influence skin temperature which was not accounted for in this analysis. The thermal gradient analysis does suggest that any differential T_C_ responses were not related to heat transfer through body surface layers, despite differences in size. However, further research is needed to fully elucidate the influence of body size on thermoregulatory mechanisms.

The two groups in our study also differed in their fitness status. Previously, it has been observed that individuals with higher fitness tend to develop heat acclimation adaptations more quickly than less fit individuals (Périard et al., 2015). In agreement with these observations, the fitter low BMI group showed enhanced T_C_ adaptations compared to the high BMI group in our study. Although the higher BMI group was exercising at a greater relative intensity (i.e., higher % of V̇O_2peak_), this does not directly equate to heat production as has been previously discussed (Cramer & Jay, 2014; Gagnon et al., 2008; Ravanelli et al., 2017; Wingo, 2015). Given the identical workload between groups in the current analysis, we would not expect differences in relative heat production (i.e., W·kg^−1^) during exercise, although heat production was not directly measured.

Despite the differential T_C_ responses seen between the low and high BMI groups, the groups had similar responses in HR and in WBSR relative to BSA. There was evidence of adaptation in these variables, without an impact of BMI, as peak HR decreased across days and relative WBSR increased by the eighth day of acclimation (Figure 1c,d). Although WBSR was not different between groups when expressed relative to BSA, the higher BMI individuals (who also had higher BSA) did have higher absolute WBSR compared to the lower BMI group. These findings contrast with previous research using a controlled‐hyperthermia protocol that showed increased HR adaptation (i.e., lower HR) in smaller individuals and increased sudomotor adaptation in larger individuals suggesting an independent influence of body size on HR and sweating responses to HA (Alkemade et al., 2021). These contrasting findings are likely related to differences in protocol design with greater thermal and cardiovascular strain induced in the controlled‐hyperthermia protocol.

Our present analysis, using 8 days of heat acclimation, may not have allowed for the higher BMI group to exhibit the same magnitude of T_C_ adaptations as the lower BMI group, as protocols of at least 14 days may be needed to see full adaptation (Périard et al., 2021). It is possible that the higher BMI individuals in the present analysis may have seen the same magnitude of adaptation as the smaller individuals if the acclimation protocol was extended past 8 days. This, in conjunction with the work discussed above (Alkemade et al., 2021), it is clear that potential time course differences and recommendations based on body size/BMI warrant future investigation.

### 4.1 Experimental considerations

There are some methodological considerations that are worth mentioning. First, the method of T_C_ measurement was not the same for all volunteers. Although the location of T_C_ measures differed between volunteers, there is good agreement in temperature measures with rectal temperature and ingested pills (Byrne & Lim, 2007; O'Brien et al., 1998), and measures from pills ingested ranging from 1 h to 12 h prior to measurement do not differ (Notley et al., 2021). Additionally, all volunteers utilized the same method across trials with pill ingestion occurring at a similar time on the evening prior to trials (±1 h), except for one volunteer who ingested a pill on Day 1 and Day 8 but inserted a pill as a suppository on Day 4 due to the ingested pill not reading. Second, we utilized a controlled work rate protocol as it is the most practical to implement for many athletic, military, and industrial groups, so our results cannot be extended to other types of heat acclimation protocols (e.g., controlled hyperthermia, clamped heart rate, etc.). Given the body mass differences between groups, we would expect a higher absolute heat production in the higher BMI group, as absolute heat production increases proportionally to body mass during weight‐bearing exercise (Marino et al., 2000; Robinson, 1942). However, heat production was not measured in the present analysis. Additionally, the expected differences in absolute heat production between groups did not lead to differences in peak T_C_ or delta T_C_ on Day 1, potentially due to differences in heat dissipation based on body size, as was discussed previously. Lastly, within this secondary statistical analysis, it was not possible to identify the contributions of other variables (BF%, fitness status) between groups, and to maintain generalizability, we sought to independently test BMI using the population cutoff for overweight individuals (BMI > 25). Given there are no standardized cutoffs for BF% or fitness (particularly considering standards over the age profile), future research should evaluate the relative importance of BF% and fitness status within the context of heat acclimation. Finally, skin temperature was evaluated in a portion of these data, but we were unable to quantify the impact of sweat evaporation on skin temperature. Future research should evaluate evaporative efficiency between groups of varying body size to fully elucidate these possible effects.

In summary, our data suggest that individuals with BMI > 25 may not show thermal (T_C_) adaptations to the same degree or as quickly as individuals with BMI < 25 during an 8‐day period of repeated exercise‐heat stress with a controlled rate of treadmill walking. Surprisingly, HR and WBSR adaptations (controlled for body size) were not different between groups, suggesting that these mechanisms do not explain the differential thermal adaptations. One possibility is that biophysical parameters (greater surface area for heat dissipation relative to mass for heat generation) allowed for greater adaptations in the smaller individuals. However, clearly more work is needed to evaluate specific mechanisms as well as longer heat acclimation protocols.

## AUTHOR CONTRIBUTIONS

Conceived and designed research: R.M.S., K.E.B., N.C., G.E.W.G. Performed experiments: S.C.B., R.M.S., K.E.B., G.E.W.G. Analyzed data: S.C.B., R.M.S., G.E.W.G. Interpreted results of experiments: S.C.B., R.M.S., K.E.B., N.C., G.E.W.G. Prepared figures: S.C.B. Edited and revised manuscript: S.C.B., R.M.S., K.E.B., N.C., G.E.W.G. Approved final version of manuscript: S.C.B., R.M.S., K.E.B., N.C., G.E.W.G.

## FUNDING INFORMATION

This research was funded by the Military Operational Medicine Research Program (grant no. MO220032, G. Giersch) and with internal institute funds (R. Salgado).

## CONFLICTS OF INTEREST

The authors declare no conflicts of interest.

## ETHICS STATEMENT

All procedures were approved by the Medical Research and Development Command Institutional Review Board, and all volunteers provided informed consent. All protocols adhered to the provisions of Code 45 of Federal Regulations Part 46 (United States of America).

## DISCLAIMER

The views, opinions, and/or findings contained in this article are those of the authors and should not be construed as an official United States Department of the Army position or decision unless so designated by other official documentation. This article is approved for public release and distribution is unlimited.

Citations of commercial organizations and trade names in this report do not constitute an official Department of the Army endorsement or approval of the products or services of these organizations.

This research was supported in part by an appointment to the Department of Defense (DOD) Research Participation Program administered by the Oak Ridge Institute for Science and Education (ORISE) through an interagency agreement between the US Department of Energy (DOE) and the DOD. ORISE is managed by ORAU under DOE contract number DE‐SC0014664. All opinions expressed in this paper are the author's and do not necessarily reflect the policies and views of the US Army, DOD, DOE, or ORAU/ORISE.

## Data Availability

Data are available from the corresponding author upon reasonable request and approval of a data‐sharing agreement.
